# The Diagnostic Properties of Medical History in the Diagnosis of Tubal Pathology among Subfertile Patients

**DOI:** 10.5402/2012/436930

**Published:** 2012-01-22

**Authors:** Egle Tvarijonaviciene, Ruta Jolanta Nadisauskiene, Kristina Jariene, Valdemaras Kruminis

**Affiliations:** Department of Obstetrics and Gynecology, Lithuanian University of Health Sciences, Eiveniu 2, 50009 Kaunas, Lithuania

## Abstract

*Objectives*. To evaluate the diagnostic performance of medical history in the diagnosis of tubal pathology among subfertile patients. *Patients and Methods*. Prospective cross-sectional study was performed. Prior to tubal evaluation, medical history data were collected. Sensitivity, specificity, and likelihood ratios (LRs) for predicting tubal pathology as determined by laparoscopy and dye test were calculated for each issue of medical history. *Results*. 39.6 % (59/149) were diagnosed with tubal pathology. The sensitivity for the different issues ranged between 1.7 and 54.2% and the specificity between 75.6 and 97.8%. The estimated highest value of positive LR is attributed to the history of ectopic pregnancy and lowest of negative LR to pelvic inflammatory disease (PID) and abdominal surgery. *Conclusion*. The positive history of PID, sexually transmitted diseases (STDs), abdominal and laparoscopic surgery, and ectopic pregnancy are satisfactory screening tests for ruling the tubal pathology in. The negative history of evaluated issues is inappropriate for ruling the tubal damage out.

## 1. Introduction

Tubal pathology is one of the main causes of infertility. It is estimated to account 12–33% [1–3]. This probably is an underestimate, since most aspects of tubal dysfunction escape our observation. Tubal disease can result from infection, endometriosis, past surgery, and tubal blockage can be at the proximal or distal portion of the tubes. Distal tubal obstruction contributes to 80% of tubal factor infertility, whereas transmural and isthmic portions or the tubes account for the rest [4]. Proximal tubal occlusion can be attributed to reversible causes such as the presence of mucus, polyps, or intramural debris [4]. Tubal disease can be accompanied by peritubal adhesions as well. In the routine fertility workup, our ability to evaluate tubal function is limited. We currently judge the degree of tubal damage mainly by tubal patency and the extent of peritubal adhesions [4], but this does not necessary equate to satisfactory function. In the routine fertility workup, tests available for evaluation of tubal function can be divided into diagnostic and screening tests [5]. The main aim of diagnostic tests is to prove pathology. Today, laparoscopy with dye (LS) is considered the best available diagnostic test for tubal factor infertility [6–8]. It is used as a reference standard in most clinical studies. LS involves hospital admission, general anesthesia and 1 to 2% complication rate including postoperative infection and injury to bowel or blood vessels, and a mortality rate of eight in 100000 [8]. Traditionally, LS is the final diagnostic procedure of any infertility investigation. Screening tests are useful in establishing the risk for tubal pathology in an individual patient. Methods used for screening purposes include patient history, hysterosalpingography (HSG), salpingosonography, and *Chlamydia trachomatis* antibody testing [5]. Physical examination is rarely helpful in detecting at risk patients for tubal disease.

The ideal screening test for the diagnosis of tubal pathology will need to be highly sensitive and specific. Sensitivity measures the number of people who truly have the disease who test positive, whereas specificity measures the number of people who do not have the disease who test negative [4]. Sensitivity and specificity can be converted into likelihood ratios (LRs). Conceptually, LRs are among the most complicated characteristics of a diagnostic test [8]. LR is a semiquantitative measure of the performance of diagnostic test which indicates how much a diagnostic procedure modifies the probability of the disease [9]. LRs assist in putting the value of testing in proper perspective. LRs are not affected by the prevalence of the disease in the population studied [5, 9]. The likelihood of a positive test result (LR+) indicates the likelihood of an abnormal test result in a patient with the disease over the likelihood of abnormal test result in a patient without the disease [5, 8]. The likelihood of negative test result (LR−) indicates the likelihood of a normal test result in a patient with the disease, over the likelihood of a normal test result in a patient without the disease [5, 8]. Calculations of LRs yield a score that allows categorization of test results [5].

 The aim of our study was to evaluate the diagnostic value of medical history and lifestyle habits in the diagnosis of tubal pathology among subfertile patients and to discuss its clinical implication.

## 2. Materials and Methods

A prospective cross-sectional study was carried out at the Department of Obstetrics and Gynecology, Kaunas University Hospital, Lithuania. Consecutive infertile women registered between May 30, 2005, through November 30, 2006, were eligible for the study. All patients initially underwent routine evaluation that included a complete medical history and physical examination, semen analysis, and hormonal assessment. Study group was selected with regard to appropriate inclusion and exclusion criteria. Inclusion criteria embraced the following: (1) infertility diagnosis according to WHO definition; (2) women's age 19–42 years; (3) confirmed ovulatory cycles and/or normal ovarian reserve; (4) absence of severe sperm pathology; (5) patient's consent to the study. Exclusion criteria were (1) previous HSG related to infertility, (2) previous diagnostic LS related to infertility, (3) previous laparoscopic or abdominal tubal surgery related to infertility, and (4) contraindications for LS. Women who fulfilled these criteria were enrolled.

Study participants were asked to complete a standardized questionnaire describing their demographic, socioeconomic data, previous medical history, and some habits of the lifestyle. A standardized data sheet was filled by the trained senior resident after the interview with the patient. As screening tests for tubal pathology, history of pelvic inflammatory disease (PID), history of sexually transmitted diseases (STD), history of intrauterine contraceptive device (IUCD), history of ectopic pregnancy, history of abdominal surgery, history of appendectomy, history of vaginal surgery, and history of laparoscopic surgery were considered. As screening tests for tubal pathology, the following factors of the lifestyle, that is, smoking, alcohol abuse (more than once per week), and four or more sexual partners throughout life, smoking and alcohol abuse and four or more sexual partners throughout life altogether were selected.

Screening for acute lower genital tract chlamydial infection by nucleic acid amplification assay was performed before diagnostic LS. LS and dye test were performed within 1–3 months after enrolment. The procedure was carried out at the Department of Obstetrics and Gynecology under the general anesthesia by one of the three investigators. *Storz *laparoscope for standardized three-puncture technique was used. A thorough inspection of the pelvis, internal genitalia, appendix, and liver region was performed, followed by testing the patency of the Fallopian tubes using methylene blue dye. Tubal status (patency or occlusion) and periadnexial adhesions were assessed by investigator blind to the data of questionnaire and interview results and recorded on a standardized operation note. Bilateral spill of dye and absence of periadnexial adhesions was considered as a normal tubal status at LS. Cases with the evidence of unilateral or bilateral proximal or distal tubal obstruction and/or periadnexal adhesions were considered as abnormal and classified as tubal pathology. Proximal tubal occlusion was diagnosed whenever the dye was injected under pressure and the dye did not fill the tube. Distal tubal occlusion was diagnosed when entire tube was filled and distended with or without ampullary dilatation but with no free spillage. Periadnexal adhesions were scored according to American Fertility Society criteria [10]. The same criteria were used for distal tubal obstruction. Revised American Fertility Society criteria were used for endometriosis [11]. In patients with only one tube, the LS was interpreted as abnormal when the remaining tube demonstrated obstruction and/or evidence of periadnexial adhesions.

Four items were the focus of the study: Patient, Index Test (medical history and lifestyle habits), Comparision Test (LS), Outcome (tubal pathology).

Sensitivity, specificity, likelihood ratios (LRs), and posttest probability for positive and negative results were calculated regarding LS as a reference standard. Tubal pathology detected by screening tests and diagnosed at LS was compared in 2 × 2 table. Confidence intervals (95% CI) were reported in order for statistical comparisons to be made.

The categorization score of the screening tests was used regarding values of LRs [5]. LR+ of 2–5 indicates a fair clinical test, 5–10 good, and >10 excellent. An LR− of 0.5–0.2 indicates a fair clinical test, 0.2-0.1 good, and <0.1 excellent. Comparisons of screening tests were made regarding values of LRs.

The prevalence of tubal infertility in women attending Department of Obstetrics and Gynecology of Kaunas University of Medicine was estimated 30% (95% CI: 22.6–37.4). Assuming estimates of sensitivity and specificity 80% and 90%, respectively, we aimed to recruit a minimum of 144 women, 48 of whom would have tubal disease diagnosed at LS. This sample size represents statistical power of 80% (*α* = 0.05, *β* = 0.8). Data were analyzed using SPSS (Statistical Package for Social Sciences, Microsoft Inc.) software version 16.0.

The study was approved by Ethics Committee of Kaunas region. The written informed consent was signed by every patient included in the study.

## 3. Results

**Figure 1:**
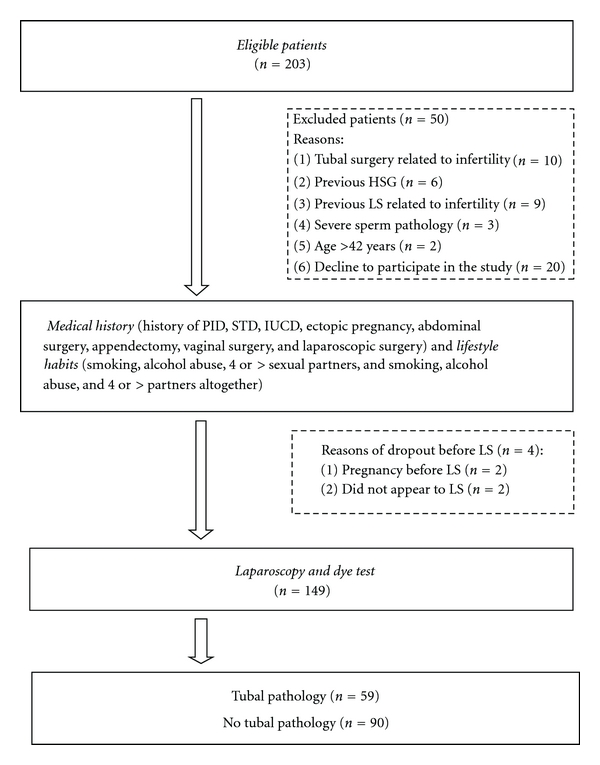
General outline of the study.

203 consecutive women were approached within the study period, and 149 of them were analyzed (Figure 1).

The mean (SD) age of study participants was 30.5 (4.2) years (range 20–41). The majority of study population were urban (69.8%) and married or lived with the partner (99.3%). 44 (29.6%) women had lower than college education, 30 (20.1%) college education, and 75 (50.3%) university degree. 74.5% of analyzed women used contraception in the past, and the most popular contraceptives were condoms and oral pills. Mean (SD) duration of infertility was 4.7 (3.5) years (range 1–18). Infertility was reported as primary and secondary by 93 (62.4%) and 56 (37.6%) women, respectively. 28.9% of study participants smoked during the study, and 91.9% were alcohol users. 10.8% of women used alcohol once per week or more often. 34.2% of analyzed patients had one sexual partner throughout life, 26.8% had 2,  20.8% had 3, and 1.1% had 4 and more. 4 patients were positive for acute lower tract chlamydial infection and received treatment with antibiotics.

39.6% (59/149) were diagnosed with tubal pathology. Tubal pathology detected at LS is shown in Table 1.

 The mean (SD) score for distal tubal occlusion according to classification of American Fertility Society was estimated at 21.1 (12.8) (range 5–46) and the mean (SD) score for periadnexial adhesions at 28.7 (21.0) (range 4–72), respectively.

Other pelvic pathologies found at LS among the study patients were minimal and mild endometriosis (stage I or II)—26.8% of cases, moderate and severe endometriosis (stage III and IV)—7.4%, polycystic ovaries—9.4%, ovarian cysts—2.7%, uterine myomas—8.1%, and uterine anomalies—3.4% of cases. Perihepatic adhesions (*Fitz-Hugh-Curtis* syndrome) were found in 7.4% of cases.

53 (35.5%) women reported a past history of PID. 17 (11,4%) reported a past history of STD that included the following: *Chlamydia* (*n* = 10), trichomonal infection (*n* = 3), genital warts (*n* = 3), human papillomavirus infection of the cervix (*n* = 3), genital herpes (*n* = 1), and syphilis (*n* = 2). 12 patients mentioned the history of one, five the history of two STDs. 10 (6.7%) women reported past history of IUCD use and 11 (7.4%) past history of ectopic (tubal) pregnancy. 43 (28.9%) patients recalled past history of abdominal surgery that included the following: lower uterine Caesarean section (*n* = 4), cystectomy (*n* = 4), adnexectomy (*n* = 3), resection of the ovary (*n* = 2), tubectomy (*n* = 1), tubectomy (*n* = 4), appendectomy (*n* = 22), hernioplastic operations (*n* = 4), laparotomies due to diverticula of the intestines (*n* = 2), peritonitis/pelvioperitonitis (*n* = 3), and other laparotomies (*n* = 4). 34 women had one, 8 had two, and 1 had three abdominal surgical procedures. 17 (11.4%) patients reported laparoscopic surgery in the past (cystectomy (*n* = 9), tubectomy (*n* = 6), tubectomy (*n* = 1), cholecystectomy (*n* = 1), and diagnostic LS because of PID (*n* = 1)), 16 of them had one and one woman had two laparoscopic operations. 36 (24.2%) participants reported past history of vaginal surgery that includes the following: large loop excision of the cervix (*n* = 6), dilatation and curettage (*n* = 15), and surgical termination of pregnancy (*n* = 18). 33 women reported one vaginal operation, and three reported two vaginal procedures. 74 (49.5%) participants reported at least one surgical procedure—laparotomic, laparoscopic, or vaginal.

The diagnostic performance of medical history and lifestyle habits in the diagnosis of tubal pathology is presented in Tables 2 and 3.

## 4. Discussion

Screening tests for tubal pathology are useful in establishing the risk for tubal factor subfertility in an individual patient. Depending on the risk estimate, decisions can be made concerning additional testing or treatment: in a low-risk patient, one may decide to postpone more extensive investigation of tubal function, whereas, in a high-risk patient, one may wish to proceed immediately to diagnostic testing. The importance of patient's history in risk assessment for tubal factor subfertility in our study was evaluated. The clinical value of the test was assessed concerning values of LR+ and LR− and changes of posttest probability for positive and negative result.

In accordance with Standards for Reporting of Diagnostic Accuracy (STARD), the screening test should be evaluated in the “independent” group of patients [12]. This was not done in our study due to ethical issues.

Tubal pathology was assessed during LS and dye test (Table 1). Three cases of unilateral and two cases of bilateral proximal tubal occlusion were identified. Because of small number, the proximal tubal obstruction was not evaluated as a separate group of pathology and disturbances of tubal patency were assessed as unilateral or bilateral tubal occlusion. The definition of tubal lesions regarding infertility differs among researches and is not uniform [13]. The chosen definition of tubal pathology as a unilateral or bilateral proximal or distal tubal obstruction and/or periadnexial adhesions embraced the whole spectrum of tubal lesions and could have an impact on the study results. The results could be influenced by several methodological characteristics of the study as well. Medical history data were collected before any therapeutic procedures by filling a standardized questionnaire. The so-called “memory” or “recall bias” of some patients could not be excluded as well as some patients might not accurately understand the emerging issues of the questionnaire—almost one third of the study participants had lower than college education. Medical history data collection by filling questionnaires was criticized by several authorities [14–16]. There is less possibility of the mistakes and greater possibilities for the clarification of some uncertainties during the direct interview [16]. This is of particular importance for the completion of the information concerning STDs and habits of the lifestyle. To provide this history the high trust between the patient and the interviewer is obligatory. The direct interview with the patient concerning the issues of the questionnaire performed by the trained senior resident in our study minimized the possibility of those mistakes.

The relationship between the previous PID and tubal pathology was thoroughly discussed in scientific papers [17–21]. The increased risk for tubal factor infertility for the patients with the history of PID was evaluated by numerous researches [20, 22–25] and summarized in systematic review [26]. Nevertheless, the studies on the diagnostic value of the history of PID as a screening test for prediction of tubal lesions are limited [22]. Usually low sensitivities were reported by the investigators. The sensitivity of 11% and specificity of 97% of the history of PID was estimated by Logan et al. [27]. These estimates in the study of Johnson et al. were 25% and 75%, respectively [28]. The diagnostic properties closest to our findings (Table 2) were estimated by Hubacher et al. (sensitivity of 47% and specificity of 50%) [29]. This latter study was criticized by the opponents because of final conclusions [14–16]. The estimated value of LR+ of the history of PID in our study (Table 2) means that normal pretest probability of 30% after the positive history would change to 49.6% and this change of risk for tubal lesions would be statistically significant. From this point, the positive history of PID could be assessed as a fair diagnostic test for ruling in the diagnosis of tubal pathology. On the other hand, the estimated LR− value of 0.6 would convert the normal pretest probability of 30% to 20.5% after the negative test and this change of risk would not be statistically significant. So the negative history of PID is insufficient test for ruling out the diagnosis of tubal pathology. Several moments should be discussed when assessing the clinical performance of the history of PID in the diagnosis of tubal pathology in our study. First, the “silent” course of PID [30, 31] when the tubal damage could be sustained without the apparent knowledge of the women. On the other hand, sometimes to liberal treatment with antibiotics without the clear evidence of infection could mislead some patients. And, finally, the other reasons not related to infection like previous surgery (Table 2), endometriosis (moderate and severe endometriosis was found in 7.4% of cases), or nonspecific reasons leading to proximal tubal occlusion could cause the tubal damage [4].

The estimated relatively low sensitivity for the history of STDs in our study (Table 2) corresponds to the estimates of Logan et al. [27]. The similar data were reported by Johnson et al. in the evaluation of diagnostic possibilities of the history of cervical neoplasia associated with HPV infection [28]. There were 10 cases of the history of chlamydial infection and 2 cases of syphilis in our study, but the cases with gonococcal infection were lacking. However, chlamydial and gonococcal infection in particular is strongly associated with tubal damage [32], and this issue was supported by the data of meta-analysis [26]. The clinical manifestation of STDs has transformed during the several last decades. Syphilis could mimic a variety of illnesses from tumor to psychosis, gonorrhea often manifest as membranous mucopurulent discharge or even arthritis, and *Chlamydia* is associated with subclinical salpingitis. Changes of the susceptibility to antibiotics as well sometimes lead to the difficulties of the diagnosis and management of STDs. The estimated LR+ of the history of STDs in our study allowed to assess a positive test as a fair diagnostic test in ruling in the tubal pathology (Table 2). But calculated LR− of 0.9 makes this test inappropriate for ruling the tubal damage out.

 Only 10 patients of the study group were IUCD users. All of them suffered from secondary infertility. The calculated low sensitivity and LRs values (Table 2) classified the history of IUCD as inappropriate screening test for ruling in and ruling out the tubal pathology, that is, the change of the risk for tubal damage after positive and negative test result would be insignificant. Similar diagnostic properties of the history of IUCD were estimated by Logan et al. [27]. In overall, the data on the history of IUCD use in relation to tubal pathology are controversial. Reports by Forman et al. and Cramer et al. supported the issue about elevated risk of tubal damage in IUCD user's [17, 33], and other studies denied this relationship [29, 34]. However, the recently published meta-analysis still showed a mild association between IUCD use and tubal pathology [26].

 Ectopic pregnancy according to the published data had a strong correlation with tubal damage [20, 24, 35, 36]. This relationship was proved in the systematic review [26]. Nevertheless, the data on the diagnostic properties of the history of ectopic pregnancy in the prediction of tubal damage were lacking. 11 cases of ectopic pregnancy were found in our study group. All cases were surgically treated tubal pregnancies. According to the values of LRs, the positive history of ectopic could be classified as a good screening test for ruling in the tubal pathology (Table 2) because of high posttest probability for the tubal lesion. However, too high LR− made this test unsatisfactory for ruling out the tubal disease because of unchanged risk for tubal pathology after negative test.

 The data on the diagnostic properties of the history of previous surgery in the diagnosis of tubal pathology were scarce. Logan et al. estimated insufficient diagnostic performance of the past history of lower abdominal surgery and obstetric- and/or gynaecology-related procedures [27], whereas, regarding data of Johnson et al., the previous pelvic surgery could be assessed as a fair clinical test for ruling in the tubal damage [28]. The same study indicated that the diagnostic value of the history of appendectomy was an inappropriate screening test. More information could be found regarding the causal relationship between the pelvic/abdominal operative procedures and elevated risk of tubal damage [17, 20, 24, 34]. Several studies evaluated the controversial issues on the influence of complicated and uncomplicated appendicites and appendectomy [37–39]. The recent systematic review summarized that previous appendectomy indicated a mildly increased risk of tubal disease with common odds ratio of 2.0 in case-control studies, but no increased risk in the cohort studies [26]. The situation with pelvic surgery was opposite—the increased risk of tubal pathology was proved by pooled cohort studies with odds ratio 3.6 but not by the case-control studies (OR 1.5, 95% CI 0.2–11.6) [26]. Results of our study demonstrate comparatively low sensitivity and high specificity of previous abdominal surgery, appendectomies, and vaginal and laparoscopic surgery (Table 2). Cases of abdominal surgery embraced the cases of appendectomies, and laparoscopic approach for appendectomies in our study was not used. Cases of complicated and uncomplicated appendicitis were not separated. Laparoscopic interventions included cases of tubal ectopics (*n* = 6). According to the results of our study, the history of abdominal or laparoscopic surgery could be assessed as fair screening tests in ruling the tubal pathology in. The history of appendectomy reached the value of LR+ of 5.2 and could be attributed to the group of good diagnostic tests in ruling the tubal damage in (Table 2). However, too high values f LR− make those tests inappropriate for ruling the diagnosis of tubal damage out (Table 2).

 The impact of lifestyle habits on general reproductive performance was recently summarized in the systematic review [40]. Multiple sexual partners increased the risk of PID and STDs [41, 42]. The data of causal relationship between tubal pathology and lifestyle habits are scarce and controversial. There is evidence concerning relations of smoking and tubal pathology [43] as well as alcohol abuse and multiple sexual partners and tubal disease [34]. Some of those issues are not supported by other researches [44]. The diagnostic value of the lifestyle habits in the diagnosis of tubal pathology was not analyzed. According to our calculations, the diagnostic properties of the history of lifestyle habits are insufficient to rule the tubal pathology in or to rule it out (Table 3). The posttest probability of tubal damage after positive and negative result remains unchanged (Table 3).

## 5. Conclusions

The positive history of previous PID, STD, and abdominal and laparoscopic surgery can be qualified as a fair screening tests to rule the tubal pathology in. However, the negative history of PID, STD, and abdominal and laparoscopic surgery is inappropriate tests to rule the tubal pathology out. The positive history of ectopic pregnancy and appendectomy shows to be attributed to the good screening tests in ruling the tubal disease in, but the negative history of ectopic pregnancy and negative history of appendectomy are insufficient to rule the tubal pathology out. The history of IUCD, vaginal surgery, and lifestyle habits has insignificant value in the diagnosis of tubal pathology among subfertile patients.

## Figures and Tables

**Table 1:** Laparoscopic findings regarding tubal pathology.

Tubal damage	*N* (%)
Unilateral proximal occlusion	3 (2.0)
Bilateral proximal occlusion	2 (1.3)
Unilateral distal obstruction	22 (14.8)
Bilateral distal obstruction	16 (10.7)
Combined unilateral proximal occlusion and contralateral distal obstruction	1 (0.7)
Periadnexial adhesions (with/without tubal occlusion)	52 (34.9)
No occlusion and no periadnexial adhesions	90 (60.4)

Total	149 (100)

**Table 2:** The diagnostic performance of medical history in the diagnosis of tubal pathology.

Medical history	Tubal pathology (*n* = 59)	No tubal pathology (*n* = 90)	Sensitivity (%) (95% CI)	Specificity (%) (95% CI)	LR+ (95% CI)	LR– (95% CI)	Posttest probability for positive result (%) (95% CI)	Posttest probability for negative result (%) (95% CI)
PID								
+	32	21	54.2	76.7	2.3	0.6	49.6	20.5
–	27	69	(41.5–66.9)	(67.9–85.4)	(1.5–3.6)	(0.4–0.8)	(41.6–57.6)	(13.9–26.9)
STD								
+	10	7	17.0	92.2	2.2	0.9	48.6	27.9
–	49	83	(7.4–26.4)	(86.7–97.8)	(0.9–5.4)	(0.8–1.1)	(40.6–56.6)	(20.7–35.1)
IUCD								
+	5	5	8.5	94.4	1.5	1.0	39.2	30.1
–	54	85	(1.4–15.6)	(89.7–99.2)	(0.4–5.0)	(0.9–1.1)	(31.4–47.0)	(22.7–37.5)
Ectopic pregnancy								
+	9	2	15.2	97.8	6.9	0.9	74.8	27.9
–	50	88	(6.1–24.4)	(94.7–100.8)	(1.5–30.7)	(0.8–1.0)	(67.8–81.8)	(20.7–35.1)
Abdominal surgery								
+	31	12	52	86.7	3.9	0.6	62.6	20.5
–	28	78	(39.8–65.3)	(79.6–93.7)	(2.2–7.0)	(0.4–0.7)	(54.8–70.4)	(14.0–27.0)
Appendectomy								
+	17	5	28.8	94.4	5.2	0.8	69.1	25.6
–	42	85	(17.3–40.4)	(89.7–99.2)	(2.0–13.3)	(0.6–0.9)	(61.7–76.5)	(18.6–32.6)
Vaginal surgery								
+	18	18	30.5	80.0	1.5	0.9	39.2	27.9
–	41	72	(18.8–42.3)	(71.7–88.3)	(0.9–2.7)	(0.7–1.1)	(31.4–47.0)	(20.7–35.1)
Laparoscopic surgery								
+	12	5	20.3	94.4	3.7	0.8	61.4	25.6
–	47	85	(10.1–20.6)	(89.7–99.2)	(1.4–9.9)	(0.7–1.0)	(53.4–69.2)	(18.6–32.6)

**Table 3:** The diagnostic accuracy of lifestyle habits in the diagnosis of tubal pathology.

The lifestyle habit	Tubal pathology	No tubal pathology	Sensitivity (%) (95% CI)	Specificity (%) (95% CI)	LR+ (95% CI)	LR− (95% CI)	Posttest probability for positive result (%) (95% CI)	Posttest probability for negative result (%) (95% CI)
Smoking								
+	21	22	35.6	75.6	1.5	0.9	39.2	27.9
–	38	68	(23.4–47.8)	(66.7–84.4)	(0.9–2.4)	(0.7–1.1)	(31.4–47.0)	(20.7–35.1)
Alcohol abuse*								
+	5	11	8.5	87.8	0.7	1.0	23.1	30.1
–	54	79	(1.4–15.6)	(81.0–94.5)	(0.3–1.9)	(0.9–1.2)	(16.3–29.9)	(22.7–37.5)
4 or more sexual partners								
+	12	15	20.3	83.3	1.2	1.0	34.4	30.1
–	47	75	(10.1–30.6)	(75.6–91.0)	(0.6–2.4)	(0.8–1.1)	(26.8–42.0)	(22.7–37.5)
Smoking and alcohol abuse and 4 or more sexual partners								
+	1	2	1.7	97.8	0.8	1.0	25.6	30.0
–	58	88	(–1.6–5.0)	(94.7–100.8)	(0.07–8.2)	(1.0–1.1)	(18.6–32.6)	(22.6–37.4)

*The use of alcohol not less than once per week.
